# Inhibition of EZH2 ameliorates bacteria-induced liver injury by repressing RUNX1 in dendritic cells

**DOI:** 10.1038/s41419-020-03219-w

**Published:** 2020-12-01

**Authors:** Yanan Wang, Qiwei Wang, Bei Wang, Yuting Gu, Hongshuang Yu, Wanlin Yang, Xiaohui Ren, Fengtao Qian, Xiaonan Zhao, Yichuan Xiao, Yanyun Zhang, Min Jin, Meiling Zhu

**Affiliations:** 1 Shanghai Institute of Immunology, Shanghai Jiao Tong University School of Medicine, Shanghai, China; 2 CAS Key Laboratory of Tissue Microenvironment and Tumor, Shanghai Institute of Nutrition and Health, Chinese Academy of Sciences, Shanghai, China; 3 Department of Stomatology, Renji Hospital, Shanghai Jiao Tong University School of Medicine, Shanghai, China; 4 Institutes for Translational Medicine, Soochow University, Suzhou, China; 5 Department of Oncology, Xinhua Hospital, Shanghai Jiao Tong University School of Medicine, Shanghai, China

**Keywords:** Acute inflammation, Liver diseases, Acute inflammation

## Abstract

Fulminant hepatic failure (FHF) is a clinical syndrome characterized by a sudden and severe impairment in liver function. However, the precise mechanism of immune dysregulation that is significant to FHF pathogenesis remains unclear. Enhancer of zeste homolog 2 (EZH2) has been implicated in inflammation as a regulator of immune cell function. In this study, we investigated the role of EZH2 in an animal model of human FHF induced by *Propionibacterium acnes* (*P. acnes*) and lipopolysaccharide (LPS). We demonstrated that EZH2 depletion in dendritic cells (DCs) and pharmacological inhibition of EZH2 using GSK126 both significantly ameliorated liver injury and improved the survival rates of mice with *P. acnes* plus LPS-induced FHF, which could be attributed to the decreased infiltration and activation of CD4^+^ T cells in the liver, inhibition of T helper 1 cells and induction of regulatory T cells. The expression of EZH2 in DCs was increased after *P. acnes* administration, and EZH2 deficiency in DCs suppressed DC maturation and prevented DCs from efficiently stimulating CD4^+^ T-cell proliferation. Further mechanistic analyses indicated that EZH2 deficiency directly increased the expression of the transcription factor RUNX1 and thereby suppressed the immune functions of DCs. The functional dependence of EZH2 on RUNX1 was further illustrated in DC-specific *Ezh2*-deficient mice. Taken together, our findings establish that EZH2 exhibits anti-inflammatory effects through inhibition of RUNX1 to regulate DC functions and that inhibition of EZH2 alleviates *P. acnes* plus LPS-induced FHF, probably by inhibiting DC-induced adaptive immune responses. These results highlight the effect of EZH2 on DCs, serving as a guide for the development of a promising immunotherapeutic strategy for FHF.

## Introduction

Fulminant hepatic failure (FHF), also known as acute liver failure, is characterized by a sudden and severe impairment of liver function, which develops secondary to infection, toxin exposure, or immune-mediated attack and is a rare but potentially fatal disease^1–3^. The global mortality rate is very high; however, there are no definitive therapeutic strategies for FHF other than liver transplantation, and the mechanisms leading to this dramatic and challenging syndrome remain to be elucidated. Inflammatory responses are involved in the pathophysiology of hepatic cell death and liver injury, and they are also associated with hepatic regeneration failure. Hence, suppression of hepatic inflammation and excess tissue injury may represent a potential approach to limit the pathology of FHF.

Mice injected with heat-killed *Propionibacterium acnes* (*P. acnes*) followed by lipopolysaccharide (LPS) are one of the most commonly used animal models of fulminant hepatitis^4–7^. This model can be classified into two different phases: the priming phase, in which the injection of *P. acnes* generates granulomas, and the elicitation phase, in which LPS activates granuloma-forming cells, leading to severe liver injury. Dendritic cells (DCs), as antigen-representing cells, play a key role in the initiation of immune responses^8^. They have been suggested to play an active role in the development of liver failure, and the distribution and number of DCs reflect the progression of liver failure^9^. Moreover, DCs have been implicated in the control of acute liver injury^10–12^. Our previous data indicate that *P. acnes*-induced granuloma formation involves a DC-induced adaptive response^13^. We and others have demonstrated the critical roles of DCs in liver injury^14–16^, and we previously clarified that the DC-induced adaptive response participates in *P. acnes* plus LPS-induced liver injury^13,14^. The recruitment of DCs to the liver is a prerequisite for liver injury in this model. In the priming phase of *P. acnes* plus LPS-induced FHF, DCs and T cells cluster in the liver, leading to further proliferation and polarization of CD4^+^ T cells. DC precursors are recruited into the circulation by *P. acnes* administration, migrate into the perisinusoidal space and differentiate into mature DCs migrating to the portal area to interact with T cells and initiate T cell-mediated immune responses^13,17^. Therefore, inhibition of inflammatory infiltration and immune responses initiated by DCs would be beneficial for the treatment of FHF.

Epigenetic mechanisms are known to play crucial roles in the development and functions of DCs^18,19^. Enhancer of zeste homolog 2 (EZH2) is a core catalytic subunit of polycomb repressive complex 2 (PRC2), which mediates gene silencing by catalyzing the trimethylation of lysine 27 on histone H3 (H3K27me3) within the gene promoter region^20,21^. EZH2 has been identified to play essential roles in immune regulation and many diseases. EZH2 has been shown to control B cell development and function^22,23^ and regulate the differentiation of CD4^+^ T cells into both Th1 and Th2 cells, as well as CD4^+^ T-cell plasticity^24^. We and others previously reported the pivotal roles of EZH2 in regulating the T-cell response and emphasized this protein as a novel target for immunotherapy^25,26^. EZH2 was identified as an important regulator of macrophage activation and autoimmune inflammation^27^. EZH2 is also involved in many liver diseases and contributes to pathogenesis^28,29^. Whether targeting EZH2 is an effective treatment for FHF and the underlying mechanisms remain elusive.

EZH2 regulates the integrin signaling and adhesion dynamics of neutrophils and DCs^30^, and the majority of cases of histiocytic and dendritic cell neoplasms show strong EZH2 expression^31^. More recently, it was shown that EZH2 plays a role in regulating the activation of DCs participating in the epigenetic mechanism of allergen immunotherapy^32^. Therefore, we hypothesized that the role of EZH2 in controlling DC functions may have a profound effect on the treatment of disease. In this study, using the *P. acnes* plus LPS-induced FHF model, we uncovered that deficiency in EZH2 or inhibition of EZH2 histone methyltransferase activity significantly ameliorated liver injury and improved the survival rates of mice subjected to *P. acnes* plus LPS-induced FHF, which could be attributed to decreased infiltration and activation of CD4^+^ T cells in the liver, inhibition of T helper 1 cells and induction of regulatory T cells. We also provide molecular evidence that EZH2 suppresses the transcription factor RUNX1 to regulate DC maturation and liver injury.

## Materials and methods

### Mice

C57BL/6 mice were purchased from the Shanghai Laboratory Animal Center of the Chinese Academy of Sciences (Shanghai, China). *Ezh2*-floxed mice^27^ were crossed with *Cd11c*-Cre mice originally obtained from The Jackson Laboratory (Bar Harbor, ME, USA) to generate offspring with *Ezh2*-deficient DCs (*Ezh2*^f/f^ *Cd11c*-Cre; termed *Ezh2*^D−/−^). All mice were maintained in a specific pathogen-free facility, and all animal experiments were conducted in accordance with protocols approved by the institutional Biomedical Research Ethics Committee, Shanghai Institutes for Biological Sciences, Chinese Academy of Sciences.

### Induction of liver injury

Female wild-type (WT) and *Ezh2*^D−/−^ C57BL/6 mice (8–10 weeks old) were injected intravenously with 1 mg of heat-killed *P. acnes* suspended in 100 μL of phosphate-buffered saline (PBS). For survival analysis, mice were injected intravenously with 1 μg of LPS in 100 μL of PBS on day 7 after *P. acnes* priming. For the indicated experiments, 50 mg/kg Ro 5-3335 (Target Mol, Wellesley Hills, MA, USA), 25 mg/kg GSK126 (Selleckchem, Houston, TX, USA) or DMSO (Sigma-Aldrich, St. Louis, MO, USA) was injected intraperitoneally on days 0, 2, 4, and 6.

### Histological analysis

Liver specimens were fixed in 4% PFA and embedded in paraffin. Deparaffinized sections (5–10 μm) were stained with hematoxylin and eosin. Semiquantitative analysis of the status of liver inflammation was performed in a blinded manner as previously described^33,34^. Briefly, the H&E-stained liver slides were scored by a pathologist in a “blinded fashion” to determine the degree of inflammation as follows: 0 = no infiltration, 1 = minimal/slight infiltration, 2 = moderate infiltration, and 3 = severe infiltration.

### Cytokine analysis

The levels of IFN-γ, IL-5, IL-17, TNF-α, and IL-6 in the serum were assessed with enzyme-linked immunosorbent assay kits (R&D Systems, Minneapolis, MN, USA) according to the manufacturer’s instructions.

### Isolation of mononuclear cells (MNCs)

Spleen and liver samples from mice were minced and filtered through nylon mesh (BD Falcon, Franklin Lakes, NJ, USA) to obtain a cell suspension. Spleen and liver MNCs were obtained from the cell suspensions by Ficoll (Fresenius Kabi Norge AS, Norway) and Percoll (GE Healthcare, Boston, MA, USA) density gradient centrifugation protocols, which were performed separately according to the manufacturer’s instructions.

### Isolation and generation of mouse DCs

To isolate liver DCs, CD11c^+^ cells were positively sorted from liver MNCs using MACS CD11c microbeads (Miltenyi Biotec, Bergisch Gladbach, Germany). For the generation of mouse DCs, mouse bone marrow cells were obtained by flushing the humerus and femur bones with PBS containing 2% FBS (Gibco, Grand Island, NY, USA). DCs were cultured in the presence of granulocyte-macrophage colony-stimulating factor (GM-CSF; 10 ng/mL) and interleukin (IL)-4 (5 ng/mL) (both from PeproTech, Rocky Hill, NJ, USA) for 5 days to induce immature DCs (immature DC induction phase). The cells were further incubated with GM-CSF and tumor necrosis factor (TNF)-α (50 ng/mL; PeproTech) on type I collagen-coated plates for 3 more days to induce mature DCs (mature DC induction phase). LPS (0.1 μg/mL) was added on day 7 to further promote DC maturation.

### Mixed lymphocyte reaction (MLR)

Generated DCs were lethally irradiated (30 Gy). Then, the DCs were cultured in graded doses with CD4^+^ T cells (3 × 10^5^ cells/well) isolated from naive BALB/c mice in RPMI 1640 medium for 5 days. [^3^H] Thymidine (1 μCi/well, Shanghai Institute of Applied Physics, Chinese Academy of Sciences, China) was added 18 h before the end of the culture period. The cells were then harvested onto glass fiber mats for measurement of [^3^H] thymidine incorporation.

### Flow cytometric analysis

Cells were stained with the following antibodies obtained from BD Biosciences (Franklin Lakes, NJ, USA), eBioscience, or BioLegend (San Diego, CA, USA): anti-CD4 (BioLegend, 100451), anti-CXCR3 (eBioscience, 12-1831-82), anti-CCR7 (eBioscience, 12-1971-82), anti-CD44 (eBioscience, 12-0441-83), anti-CD62L (BioLegend, 104406), anti-CD11c (BD Bioscience, 553801), anti-MHCII (BioLegend, 107631), anti-CD80 (BD Biosciences, 553769), anti-CD86 (BD Biosciences, 553692), anti-B220 (eBioscience, 12-0452-83), anti-CD8 (BD Bioscience, 553032), anti-CD103 (BD Bioscience, 557495), anti-CD45 (BioLegend, 103132), and anti-CD11b (BioLegend, 101263). For Th1 and Treg analyses, cells were stained for surface markers, permeabilized with the Intracellular Fixation and Permeabilization Buffer Set (eBioscience, 88-8824-00), and then stained with anti-IFN-γ (BioLegend, 505825) and anti-FOXP3 (eBioscience, 17-5773-82) antibodies. The following antibodies were obtained from Cell Signaling Technology (Danvers, Massachusetts, USA): anti-Ezh2 (#5246), anti-Runx1 (#4336), and anti-H3K27me3 (#9733). The Alexa Fluor™ 488 Goat Anti-Rabbit SFX Kit from Invitrogen (Carlsbad, CA, USA) was used as a secondary antibody. Multicolor flow cytometric analysis was performed using a CytoFLEX LX (Beckman Coulter, Indianapolis, IN, USA).

### Quantitative real-time PCR and a chromatin immunoprecipitation (ChIP) assay

Total RNA was extracted from tissues or cells at the indicated time points and subsequently reverse transcribed using the Reverse Transcription System (Takara, Shiga, Japan). Quantitative real-time PCR was performed using SYBR Green PCR mix on the ViiA 7 Real-Time PCR System (Applied Biosystems, Foster City, CA, USA). The reaction protocol used was 95 °C for 5 min, followed by 35 cycles of 95 °C for 15 s, 60 °C for 60 s, and 72 °C for 5 min. β-actin was used as an internal control to normalize differences in the amount of total RNA among the samples. ChIP was performed according to the manufacturer’s recommendations (Millipore, Billerica, MA, USA). The primers are listed in Supplementary Table 1.

### Statistical analysis

All values are presented as the mean ± S.E.M. Significant differences were evaluated using an independent-sample *t* test or the Wilcoxon rank test, and multiple treatment groups within individual experiments were compared by ANOVA or the Kruskal–Wallis test, followed by the Bonferroni posttest or Dunn’s posttest to compare differences between groups. The log-rank test was used for survival analysis. Values of *P* < 0.05 were considered significant. Sample sizes of all experiments were pre-determined by calculations derived from our experience. No sample was excluded from the analyses. Animals were not randomly assigned during collection, but the strain, sex, and age of the mice were the same, and the data analysis was single masked. Investigators were not blinded to the group allocation during the experiment and outcome assessment. The number of replicates was indicated in each figure legend.

## Results

### EZH2 expression increased in DCs in the liver during bacteria-induced FHF

**Fig. 1:**
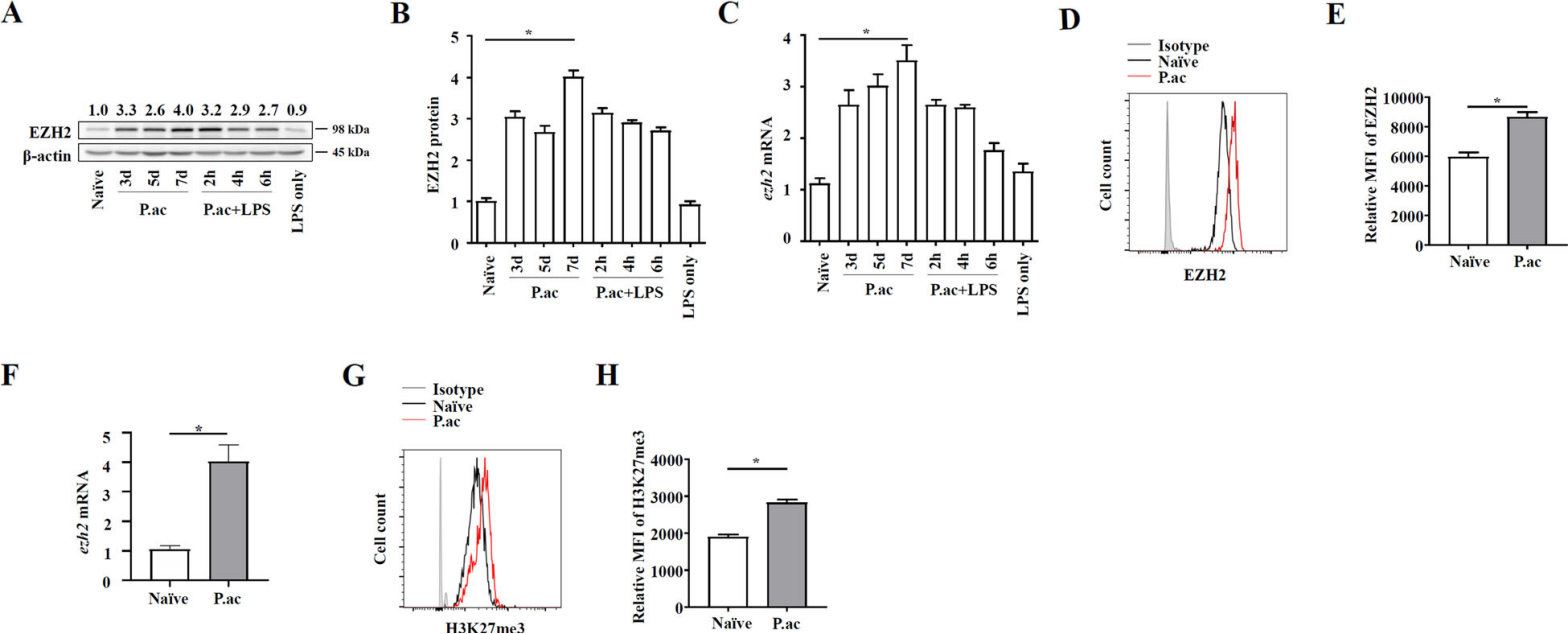
EZH2 expression increased in DCs in the liver during bacteria-induced FHF. Mice were injected with *P. acnes* (P.ac) suspended in phosphate-buffered saline (PBS). LPS was injected on day 7 to induce FHF (*n* = 8 mice per group). **A**, **B** The protein and **C** mRNA expression of EZH2 in liver tissues collected at different time points were detected by immunoblotting and QPCR. On day 7 after P.ac priming, **D**, **E** flow cytometry analysis of EZH2 expression in CD11c^high^MHCII^high^ DCs, **F** QPCR analysis of the mRNA level of EZH2 in CD11c^+^ DCs (2 × 10^5^ cells), and **G**, **H** flow cytometry analysis of H3K27me3 expression in CD11c^high^MHCII^high^ DCs were performed. Representative images from one of three experiments are shown. Data are shown as the mean±SEM of three independent experiments. *P < 0.05.

To determine the expression of EZH2 in FHF, *P. acnes* was injected intravenously into C57BL/6 mice to mimic clinical FHF. The results revealed that the EZH2 protein level in the liver increased with FHF progression and reached its highest level on day 7 (Fig. 1A, B). The trend for mRNA expression was similar to that for protein expression (Fig. 1C). To explore the role of EZH2 in DCs, we detected the expression of EZH2 and H3K27me3 in CD11c^high^MHCII^high^ DCs from the liver by flow cytometry. The results showed that both the protein and mRNA expression levels of EZH2 were increased in FHF DCs compared to control DCs (Supplementary Figs. 1A, 2A, and Fig. 1D–F). Consistently, the level of H3K27me3 was also increased (Fig. 1G, H). These results showed that EZH2 expression and methyltransferase activity were induced in FHF over time, suggesting that EZH2 might be related to the development of FHF.

### EZH2 deficiency in DCs ameliorated the severity of bacteria-induced liver injury and reduced the mortality of FHF

**Fig. 2:**
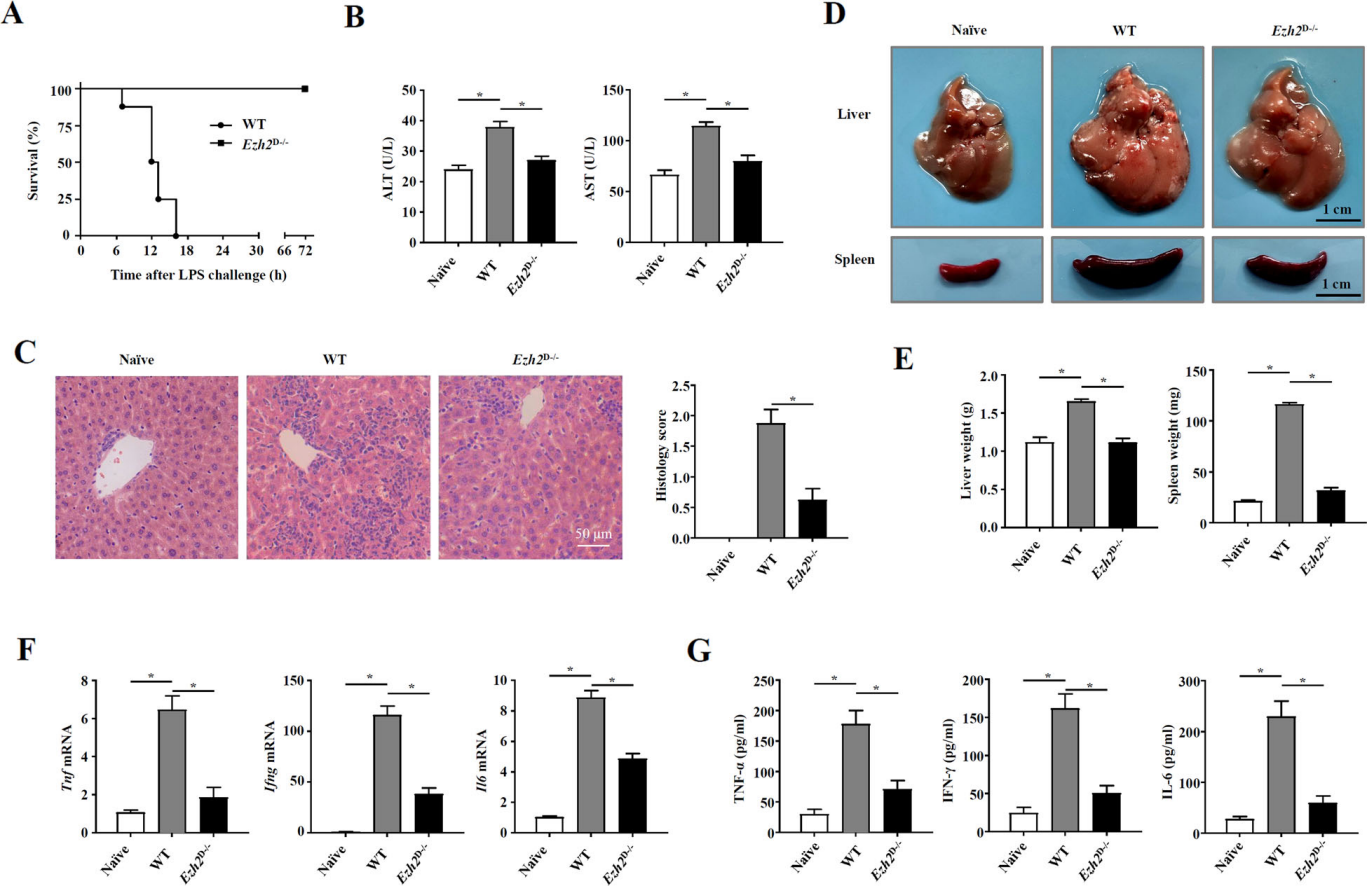
EZH2 deficiency in DCs ameliorated the severity of bacteria-induced liver injury and reduced the mortality of FHF. *Ezh2*^f/f^ (WT) mice and *Ezh2*^f/f^ *Cd11c*-Cre (*Ezh2*^D−/−^) mice were injected intravenously with P.ac suspended in PBS. *Ezh2*^f/f^ mice injected with PBS only were as a control (naive). LPS was injected on day 7 to induce FHF. **A** The cumulative survival rates of mice were analyzed (*n* = 8 mice per group) after LPS injection. On day 7 after P.ac priming, **B** the serum levels of aminotransferase (ALT) and aspartate aminotransferase (AST) (*n* = 8 mice per group) were measured, **C** H & E staining (scale bar = 50 μm) and semiquantitative analysis of inflammatory conditions in the liver are shown. **D** Liver and spleen tissues were isolated and photographed (scale bar = 1 cm). Representative images from one of three experiments are shown. **E** The weights of livers and spleens were measured (*n* = 8 mice per group), **F** QPCR analysis was performed to determine the mRNA expression levels of proinflammatory genes in the liver (*n* = 8 mice per group). **G** The levels of serum TNF-α, IFN-γ, and IL-6 were measured by enzyme-linked immunosorbent assay. Data are shown as the mean ± SEM of three independent experiments. *P < 0.05.

To further assess the role of EZH2 in DCs in FHF, we crossed *Ezh2*^f/f^ mice with mice expressing Cre recombinase from a DC-specific promoter (*Cd11c*-Cre) to generate mice with conditional deletion of EZH2 in DCs (*Ezh2*^f/f^ *Cd11c*-Cre, hereafter called *Ezh2*^D−/−^; Supplementary Fig. 3A). There were no differences in the generation or distribution of DCs between WT mice and *Ezh2*^D−/−^ mice^30^; however, EZH2 might affect the generation of DC subpopulations in the liver and spleen during FHF (Supplementary Fig. 4A, B). To investigate the function of EZH2 in regulating DCs during FHF, *Ezh2*^D−/−^ mice and their WT littermates were challenged with *P. acnes*. All of the WT mice died within 18 h post LPS injection; however, the *Ezh2*^D−/−^ mice survived for more than 72 h (Fig. 2A). Weight loss did not change in the surviving *Ezh2*^D−/−^ mice after 7 days (Supplementary Fig. 5A). Alanine aminotransferase (ALT) and aspartate aminotransferase (AST) levels in the serum were detected, and there were dramatic decreases in the ALT and AST levels of *Ezh2*^D−/−^ mice compared with those of WT mice (Fig. 2B). Histological examination showed that large nodules, severe infiltration of lymphocytes, and granuloma formation were observed in liver tissue on day 7 post-*P. acnes* priming (Fig. 2C, D), and liver and spleen weights increased considerably (Fig. 2E). Quantitative real-time PCR assays revealed that the expression of inflammatory cytokine genes, such as TNF-α, IFN-γ, and IL-6, was also elevated in livers (Fig. 2F). In contrast, *Ezh2*^D−/−^ mice displayed a normal morphology without modules, dramatically decreased infiltration of lymphocytes, reduced granuloma formation, normal liver weights, and reduced inflammatory factor levels (Fig. 2C–F). Moreover, the levels of the cytokines TNF-α, IFN-γ, and IL-6 in the serum were tested, and the results showed the same trend as the mRNA expression levels in the liver (Fig. 2G). These findings suggested that deletion of EZH2 in DCs effectively attenuated the severity of bacteria-induced liver injury and improved the survival rate of mice with FHF.

### EZH2 deficiency in DCs inhibited the CD4^+^ T-cell response during bacteria-induced liver injury

**Fig. 3:**
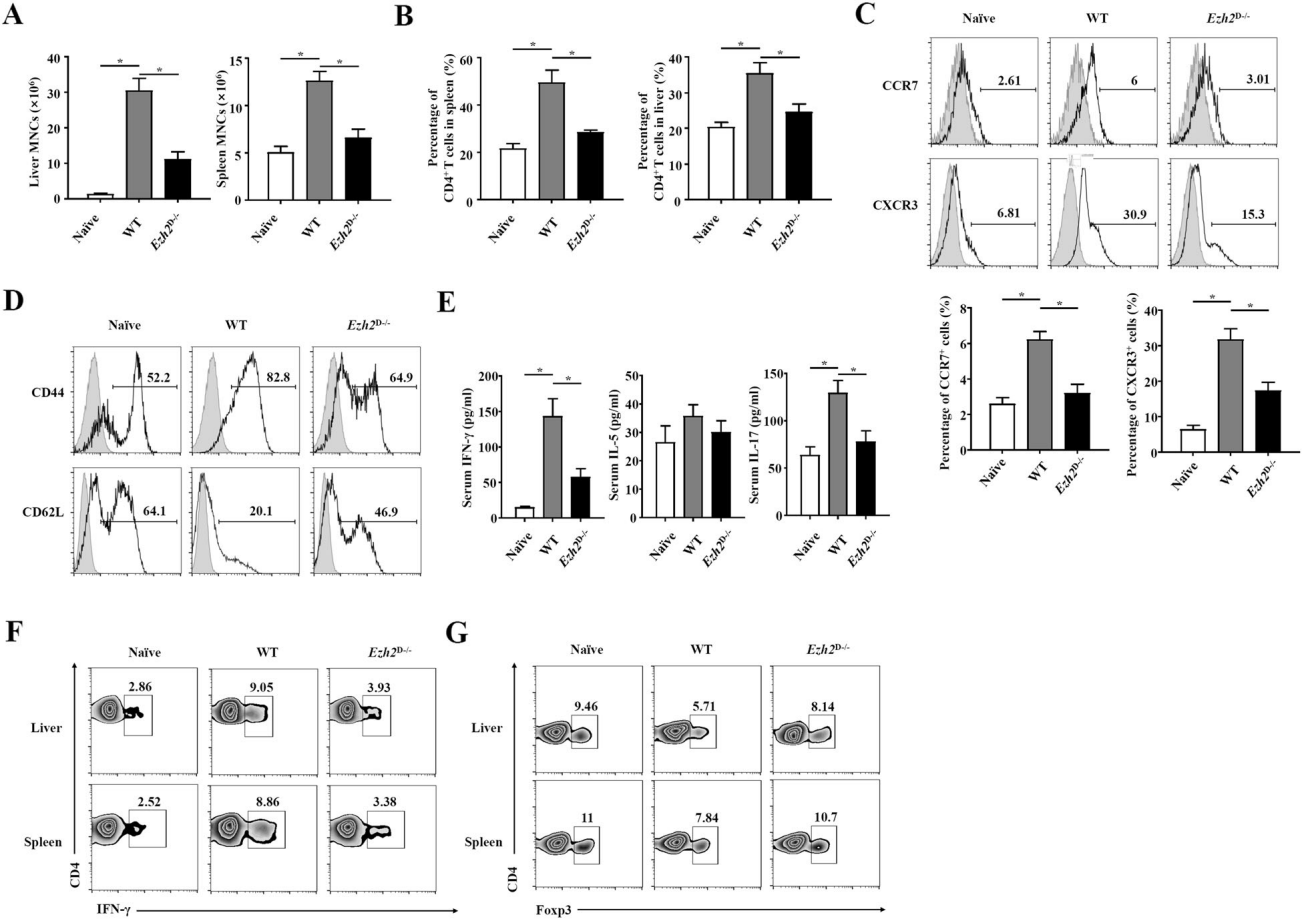
EZH2 deficiency in DCs inhibited the CD4^+^ T-cell response during bacteria-induced liver injury. *Ezh2*^D−/−^ mice and WT mice were injected intravenously with P.ac suspended in PBS. The peripheral blood, liver, or spleen were isolated from naive, *Ezh2*^D−/−^ mice and WT mice on day 7. Statistical analyses of the numbers of **A** MNCs isolated from livers and spleens and **B** the CD4^+^ T-cell percentage of MNCs are shown. **C** The levels of CXCR3 and CCR7 expressed on CD4^+^ T cells were analyzed by flow cytometry. **D** The levels of CD44 and CD62L on CD4^+^ T cells were analyzed by flow cytometry. **E** The levels of serum IFN-γ, IL-5, and IL-17 were measured by enzyme-linked immunosorbent assay (*n* = 8 mice per group). **F** The production of the inflammatory cytokine IFN-γ by CD4^+^ T cells was assessed by intracellular staining and analyzed by flow cytometry. Representative dot plots are shown. **G** MNCs isolated from livers and spleens were stained for CD4 and Foxp3 and analyzed by flow cytometry. Data are shown as the mean ± SEM of three independent experiments. *P < 0.05.

DCs elicit an inflammatory response as antigen-presenting cells by presenting antigens to CD4^+^ T cells. We analyzed the numbers of mononuclear cells (MNCs) and CD4^+^ T cells to determine whether EZH2 deficiency affects the infiltration of CD4^+^ T cells in liver injury. The results showed that the numbers of MNCs in the liver and spleen were both decreased in *Ezh2*^D−/−^ mice and that the percentage of CD4^+^ T cells in the MNC population was decreased, with the same result found in the spleen (Fig. 3A, B). Then, we analyzed the function of CD4^+^ T cells in the liver of *Ezh2*^D−/−^ and WT mice challenged with *P. acnes*. The expression of the chemokines CXCR3 and CCR7 in the liver of *Ezh2*^D−/−^ mice was considerably reduced, suggesting that the deletion of EZH2 suppressed the chemotaxis of pathogenic CD4^+^ T cells into the liver (Fig. 3C). In addition, CD4^+^ T-cell activation was suppressed in *Ezh2*^D−/−^ mice, as indicated by decreased expression of CD44 and increased expression of CD62L (Fig. 3D). We previously identified Th1 cells as central players in the pathogenesis of *P. acnes*-induced liver injury^4^. Thus, serum levels of the Th1 cytokine IFN-γ, Th2 cytokine IL-5, and additional proinflammatory cytokine IL-17 were determined. The results showed that the deletion of EZH2 significantly reduced the levels of IFN-γ and IL-17 but had no effect on IL-5 production (Fig. 3E). Intracellular staining for IFN-γ further confirmed the reduction in the IFN-γ-positive cell percentage within CD4^+^ T cells (Fig. 3F). Moreover, deletion of EZH2 increased the percentage of CD4^+^ Foxp3^+^ Tregs (Fig. 3G), although the absolute number of Tregs was not increased (Supplementary Fig. 6A). These findings suggested that the absence of EZH2 in DCs suppressed Th1 cells but promoted Treg differentiation during bacteria-induced liver injury.

### Deficiency in EZH2 inhibited the maturation of DCs

**Fig. 4:**
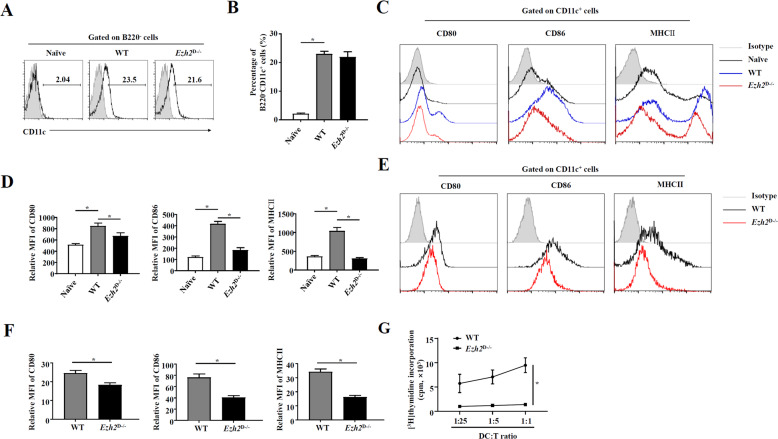
EZH2 was essential for the maturation of DCs but did not affect the recruitment of CD11c^+^ B220^−^ DC precursors. *Ezh2*^D−/−^ mice and WT mice were injected intravenously with P.ac suspended in PBS. The peripheral blood, liver, or spleen was isolated from naive, *Ezh2*^D−/−^ mice and WT mice on day 7. **A**, **B** Flow cytometry analysis of DCs was performed on day 6 to analyze CD11c and B220 expression. **C**, **D** On day 7, liver MNCs from *Ezh2*^D−/−^ mice and WT mice were assessed for CD11C, CD80, CD86, and MHCII expression by flow cytometry. **E**, **F** Bone marrow cells from *Ezh2*^D−/−^ mice and WT mice were cultured in the presence of granulocyte-macrophage colony-stimulating factor (GM-CSF) (20 ng/mL) and IL-4 (10 ng/mL) to induce DCs in vitro. On day 6, 0.1 μg/mL LPS was added to the medium, and 24 h later, DC maturation was assessed by evaluating CD80, CD86, and MHCII expression by flow cytometry. **G** DCs from *Ezh2*^D−/−^ mice and WT mice were lethally irradiated (30 Gy) and cultured in graded doses with magnetically purified CD4^+^ T cells (3 × 10^5^ cells/well) derived from the spleen of naive BALB/c mice. Five days later, T-cell proliferation was measured by monitoring the incorporation of [^3^H] thymidine. Data are shown as the mean ± SEM of three independent experiments. *P < 0.05.

In our previous studies, it was shown that circulating CD11c^+^ B220^−^ DC precursors can be mobilized to the liver, where they differentiate into mature DCs later to function in immune responses^13^. Here, we determined whether EZH2 alters the characteristics of DC precursors. As shown, the percentage of DC precursors in the peripheral blood of mice increased after *P. acnes* priming; however, *Ezh2*^D−/−^ and WT mice showed no significant difference in the percentage of CD11c^+^ B220^−^ cells (Fig. 4A, B), suggesting that EZH2 has no effect on DC precursor induction in the peripheral blood. In the liver injury model, the interactions between DCs and T cells are amplified by the continuous stimulation of liver-infiltrating CD4^+^ T cells by DCs, leading to subsequent liver injury^35^. Here, we investigated the characteristics of liver DCs. We isolated CD11c^+^ cells from the liver of *P. acnes*-induced mice and found that those from *Ezh2*^D−/−^ mice had lower expression of MHC class II (MHCII) and costimulatory molecules, including CD80 and CD86, than those from WT mice (Fig. 4C, D), indicating that deficiency in EZH2 inhibits the maturation of liver DCs. To further verify this hypothesis, we generated bone marrow-derived DCs in vitro and activated them 6 days later with LPS. The results showed that EZH2 deficiency dramatically reduced the expression of CD80, CD86, and MHCII in CD11c^+^ DCs (Fig. 4E, F). Furthermore, in a functional study, DCs generated from *Ezh2*^D−/−^ mice showed a lower capability to evoke an allogeneic mixed lymphocyte reaction, suppressing allogeneic CD4^+^ T-cell proliferation in a dose-dependent manner, than DCs from WT mice (Fig. 4G). These findings demonstrated that the absence of EZH2 did not influence the induction of DC precursors but led to reduced DC maturation.

### EZH2 repressed RUNX1 expression to regulate DC function

**Fig. 5:**
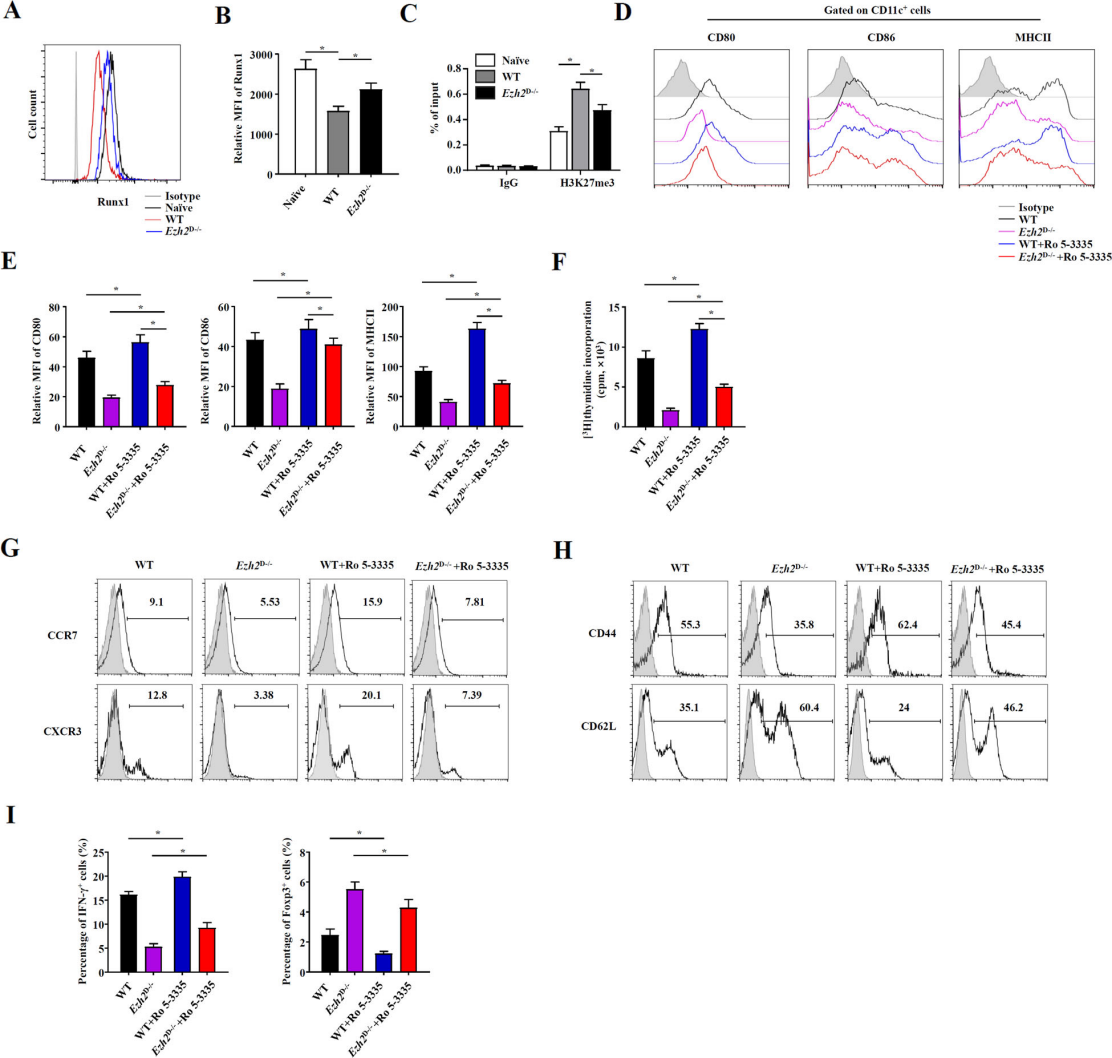
EZH2 repressed RUNX1 expression to regulate DC function. *Ezh2*^D−/−^ mice and WT mice were injected intravenously with P.ac suspended in PBS. **A**, **B** The RUNX1 protein expression in CD11c^+^ cells was analyzed by flow cytometry. **C** CD11c^+^ cells were collected for a CHIP assay specific for H3K27me3 or an IgG control. The relative amounts of H3K27me3 and IgG in regions of Runx1 were detected by QPCR. **D**, **E** Bone marrow cells from *Ezh2*^D−/−^ mice and WT mice were cultured in the presence of GM-CSF (20 ng/mL) and IL-4 (10 ng/mL) to induce DCs in vitro. On day 6, 0.1 μg/mL LPS was added to the medium and cultured with 50 μM Ro 5-3335. Twenty-four hours later, DC maturation was assessed by evaluating the surface markers CD80, CD86, and MHCII by flow cytometry. DCs generated in (**D**) were lethally irradiated (30 Gy) and cultured in graded doses with magnetically purified CD4^+^ T cells (3 × 10^5^ cells/well) derived from the spleen of naive BALB/c mice at a ratio of 1:1. Five days later, T-cell proliferation was measured by monitoring the incorporation of [^3^H] thymidine (**F**), cell chemotaxis was evaluated by detecting CCR7 and CXCR3 (**G**), cell activation was evaluated by detecting CD44 and CD62L (**H**), and proportional changes in Th1 cells and Tregs were measured by detecting the intracellular cytokines IFN-γ and Foxp3 (**I**). Data are shown as the mean ± SEM of three independent experiments. *P < 0.05.

The transcription factor RUNX1 is essential for hematopoietic stem cell formation and bone marrow progenitor differentiation, such as DC lineage differentiation. RUNX1 has also been suggested to regulate DC functions^36,37^. As EZH2 was found to interact with RUNX1 in previous research^38^, we hypothesized that EZH2 regulates DC function by interacting with RUNX1. The expression of RUNX1 in DCs isolated from *Ezh2*^D−/−^ and WT mice was detected. We found that DC-specific deficiency in EZH2 upregulated the expression of RUNX1 (Fig. 5A, B) and repressed H3K27me3 levels in the RUNX1 promoter (Fig. 5C). To explore the function of RUNX1 and its interaction with EZH2, we treated bone marrow-derived DCs generated from *Ezh2*^D−/−^ and WT mice with the RUNX1 inhibitor Ro 5-3335 and found that Ro 5-3335 treatment notably upregulated the expression of CD80, CD86, and MHCII, which was downregulated in the context of EZH2 deficiency (Fig. 5D, E). In addition, in a functional study, suppression of RUNX1 in the context of EZH2 deficiency in DCs substantially decreased the capability to evoke an allogeneic mixed lymphocyte reaction (MLR) (Fig. 5F). To further explore effects on the chemotaxis and activation of pathogenic CD4^+^ T cells, we tested the expression levels of CCR7 and CXCR3 (related to chemotaxis) and those of CD44 and CD62L (related to activation). According to the results, which were similar to those of the functional study described above, suppression of RUNX1 in the context of EZH2 deficiency in DCs substantially reduced the capabilities to activate CD4^+^ T cells and promote CD4^+^ T-cell chemotaxis (Fig. 5G, H). The proportional changes in Th1 cells and Tregs were measured by detecting the intracellular cytokines IFN-γ and Foxp3, which showed trends similar to those in the previously described assays (Fig. 5I). In addition, we examined the effects of Ro-3335 on T-cell survival and proliferation, and the results showed that there were no significant differences in the effects on T cells, suggesting that RUNX1 inhibition does not have a direct effect on T-cell function (Supplementary Fig. 7A). These results suggest that RUNX1 is suppressed by EZH2 and that EZH2 functions through regulating RUNX1 in DCs.

### Inhibition of RUNX1 ameliorated the severity of bacteria-induced liver injury in *Ezh2*^D−/−^ mice

**Fig. 6:**
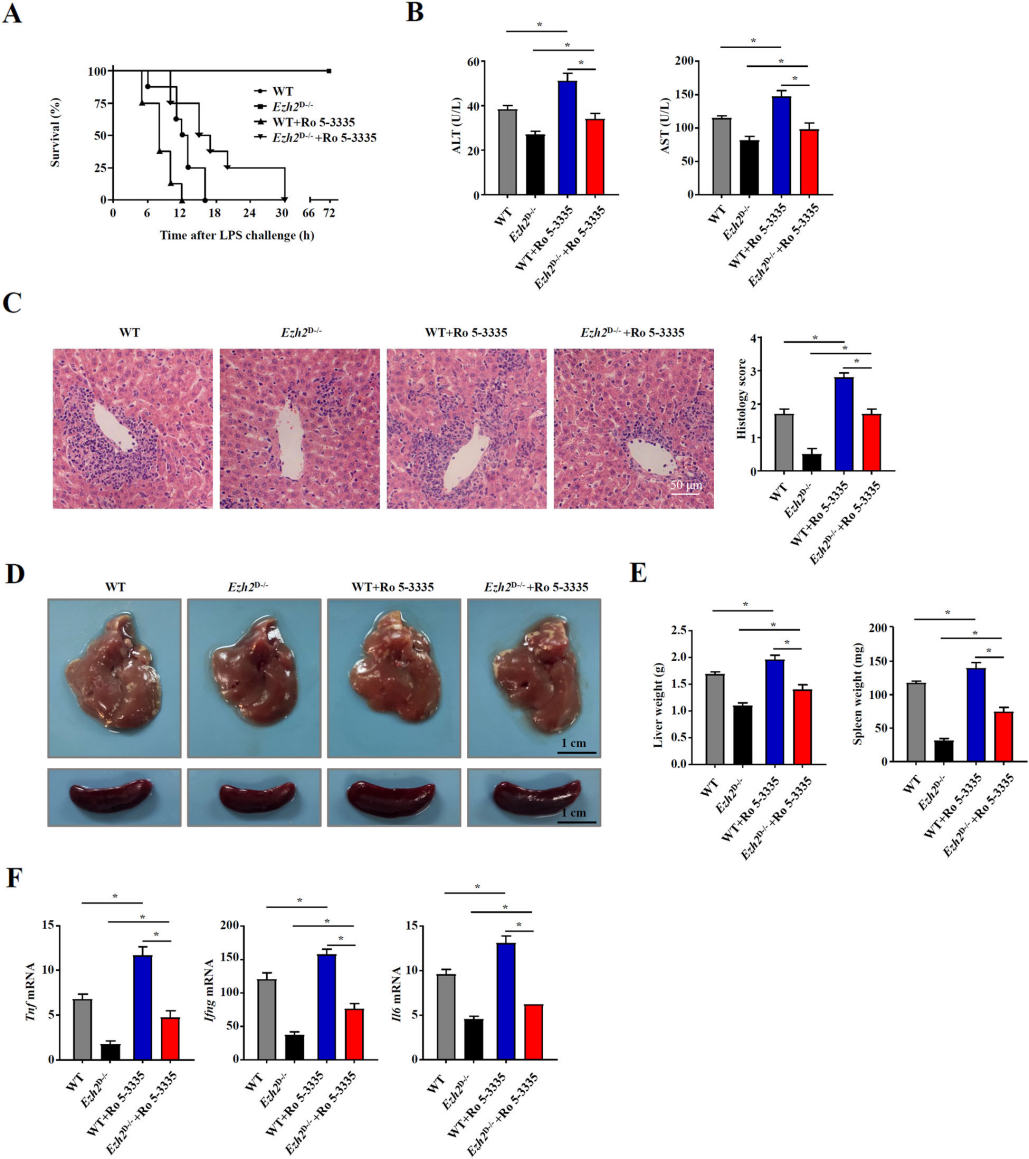
Inhibition of RUNX1 ameliorated the severity of bacteria-induced liver injury in *Ezh2*^D−/−^ mice. *Ezh2*^D−/−^ mice and WT mice were injected intravenously with P.ac suspended in PBS. DMSO or Ro 5-3335 (50 mg/kg) was injected intraperitoneally on days 0, 2, 4, and 6, and LPS was injected on day 7 to induce FHF. **A** The cumulative survival rates of mice were analyzed (*n* = 8 mice per group) after LPS injection. On day 7 after *P. acnes* priming, **B** ALT and AST levels (*n* = 8 mice per group) were measured. **C** H & E staining (scale bar = 50 μm) and semiquantitative analysis of inflammatory conditions in livers are shown. **D** Liver and spleen tissues were isolated and photographed (scale bar = 1 cm). **E** The weights of livers and spleens were measured (*n* = 8 mice per group). **F** QPCR analysis was performed to determine the mRNA expression levels of proinflammatory genes in the liver of mice (*n* = 8 mice per group). Data are shown as the mean ± SEM of three independent experiments. *P < 0.05.

To further explore the function of RUNX1 and its interaction with EZH2 in liver injury, we treated *Ezh2*^D−/−^ and WT model mice with the RUNX1 inhibitor Ro 5-3335 to examine whether RUNX1 suppression can enhance FHF. As expected, we found that all of the *Ezh2*^D−/−^ and WT mice treated with Ro 5-3335 died relatively soon after LPS injection (Fig. 6A). We also detected ALT and AST levels and found that there were dramatic decreases in these levels in *Ezh2*^D−/−^ and WT mice treated with Ro 5-3335 (Fig. 6B). Histological analysis showed that larger nodules and more severe infiltration of lymphocytes were observed in liver tissue with Ro 5-3335 treatment (Fig. 6C). Furthermore, livers isolated from *Ezh2*^D−/−^ mice treated with Ro 5-3335 had more granulomas (Fig. 6D). The weights of livers and spleens were also detected, and those from *Ezh2*^D−/−^ mice treated with Ro 5-3335 were heavier than those from mice only primed with *P. acnes* (Fig. 6E). Moreover, the mRNA expression of inflammatory cytokine genes, such as TNF-α, IFN-γ, and IL-6, was elevated in the liver (Fig. 6F). Taken together, these findings suggest that EZH2 interacts with RUNX1 to regulate DC function during bacteria-induced liver injury and that suppression of RUNX1 enhances the severity of bacteria-induced liver injury.

### The EZH2 inhibitor GSK126 protected mice from bacteria-induced liver injury

**Fig. 7:**
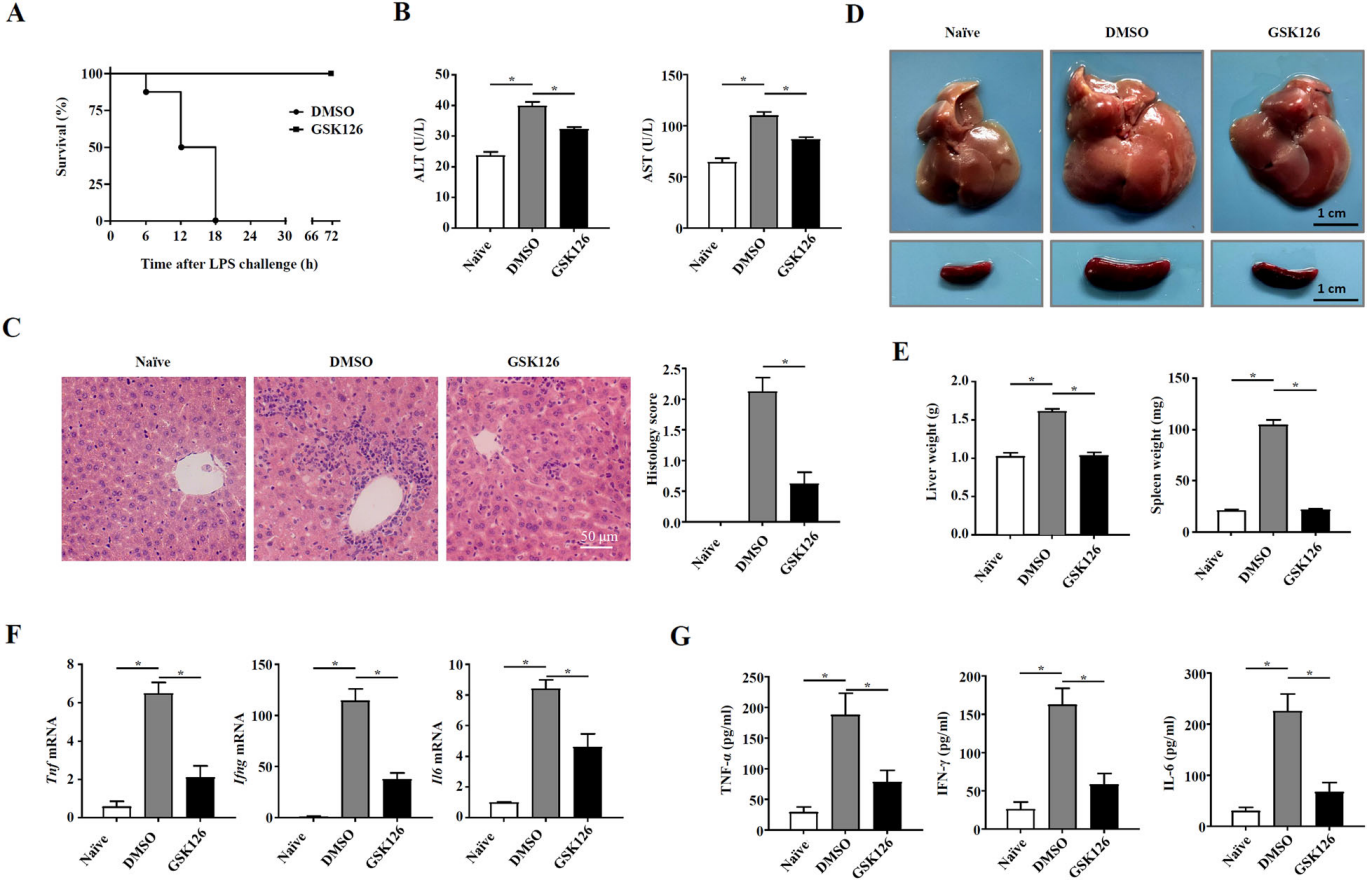
The EZH2 inhibitor GSK126 protected mice from bacteria-induced liver injury. C57BL/6 mice were injected intravenously with P.ac suspended in PBS. DMSO or GSK126 (25 mg/kg) was injected intraperitoneally on days 0, 2, 4, and 6, and LPS was injected on day 7 to induce FHF. **A** The cumulative survival rates of mice were analyzed (*n* = 8 mice per group) after LPS injection. On day 7 after *P. acnes* priming, **B** ALT and AST levels (*n* = 8 mice per group) were measured, **C** H & E staining (scale bar = 50 μm) and semiquantitative analysis of inflammatory conditions in livers are shown. **D** Liver and spleen tissues were isolated and photographed (scale bar = 1 cm) (*n* = 8 mice per group). **E** The weights of livers and spleens were measured (*n* = 8 mice per group). **F** QPCR analysis was performed to determine the relative mRNA expression levels of proinflammatory genes in the liver of mice (*n* = 8 mice per group). **G** The levels of serum TNF-α, IFN-γ, and IL-6 were measured by enzyme-linked immunosorbent assay. Data are shown as the mean ± SEM of three independent experiments. *P < 0.05.

Recently, several highly selective small-molecule inhibitors targeting EZH2, such as GSK126, have been developed. To test the effect of an EZH2 inhibitor on liver injury, GSK126 was administered intraperitoneally to C57BL/6 mice on days 0, 2, 4, and 6 after *P. acnes* priming. Then, LPS was injected on day 7, and the survival rate of the mice was investigated. We found that all mice receiving GSK126 treatment survived for 72 h, while the control mice died within 18 h, suggesting that GSK126 treatment can dramatically promote survival (Fig. 7A). Then, we analyzed ALT and AST levels and found that they were significantly lower in GSK126-treated mice (Fig. 7B). Consistent with the improved survival rate of mice, histology related to liver injury and associated inflammatory factors was dramatically ameliorated in the GSK126-treated group (Fig. 7C–F). Moreover, the levels of the cytokines TNF-α, IFN-γ, and IL-6 in the serum were tested, and the results showed the same trends as the mRNA expression levels in the liver (Fig. 7G). These results suggested that treatment with the EZH2 inhibitor GSK126 successfully inhibited *P. acnes*-induced liver inflammation and ultimately prevented liver injury.

## Discussion

This study aimed to investigate the role of EZH2 in the immune functions of DCs and its epigenetic mechanism in FHF. Here, we have shown that EZH2 deficiency established by gene knockout or inhibition of EZH2 with a small-molecule inhibitor impairs the immune responses of monocyte-derived and liver-resident DCs, which corresponds with the amelioration of bacteria-induced liver injury, increasing the survival rate of mice subjected to *P. acnes* plus LPS-induced FHF. Mechanistically, inhibition of EZH2 enhanced the expression of RUNX1 in DCs. This is the first study using EZH2 deficiency in conditional gene knockout mice to accurately explore the role of EZH2 in FHF.

Accumulating evidence suggests that EZH2 actively regulates inflammation. EZH2 is important in mediating the formation of H3K27me3 associated with gene transcription suppression^39^. It has been shown to exert essential roles in T-cell differentiation and polarization to promote the immune response in alloimmunity, act as a macrophage lineage-specific mediator of autoimmune inflammation and control immune responses in inflammatory bowel disease by affecting the development of MDSCs^24,27,40^. *P. acnes-*induced liver injury is reported to be attributed to Th1 cell-mediated inflammatory responses. In the present study, EZH2 and H3K27me3 levels were upregulated as liver injury progressed, suggesting that they are involved in liver failure by regulating immune responses. Epigenetic regulation has emerged as one of the key mechanisms regulating DC development and function. A histone deubiquitinase, Mysm1, has been reported to govern DC differentiation by modulating histone modifications and mediating recruitment of the transcription factor PU.1^18^. In the current study, we observed that the expression of EZH2 and H3K27me3 increased in DCs during liver injury. This experimental evidence raises the possibility that EZH2 is involved in liver failure by regulating the development and function of DCs.

We demonstrated that genetic deletion of EZH2 in DCs or inhibition of its enzymatic activity substantially ameliorated liver injury by inhibiting the immune responses evoked by DCs. A recent study showed that EZH2 promotes the extravasation and motility of DCs and neutrophils under inflammatory conditions^30^. Moreover, EZH2 regulates Langerhans cell transmigration, and EZH2-deficient dermal DCs exhibit increased tolerogenicity^41^. In this study, the number of CD11c^+^ B220^−^ DCs in the liver showed no significant difference between *Ezh2*^D−/−^ mice and control mice, indicating that EZH2 deficiency does not affect the recruitment of DCs to the liver. The ability of DCs to stimulate T cells is related to DC maturation. In this study, EZH2 deficiency significantly reduced the expression of CD80, CD86, and MHCII in liver DCs and monocyte-derived DCs, and these DC populations showed reduced potency in stimulating CD4^+^ T-cell proliferation. These findings suggest that EZH2 deficiency suppresses the phenotypic and functional activation of DCs. Although recent research provides experimental evidence indicating that the phenotypic and functional activation of moDCs was significantly suppressed by EZH2 inhibition with the inhibitor 3 Deazaneplanocin A (DZNep)^32^, we directly confirmed the effect of EZH2 on DCs in *Ezh2*^D−/−^ mice. According to the results of DC subset analysis during FHF, EZH2 expression correlated with the generation and distribution of DC subsets in this disease, especially DCs from the spleen, a lymphoid organ. The underlying mechanisms will be further explored in prospective studies.

EZH2 functions depend on interactions with proteins, such as AKT, AP-1, and CDK^42–44^. We previously found that EZH2 could regulate the DNA damage-responsive protein Ku80^45^ and identified the EZH2-Hsp90 interaction as an essential mechanism underlying T-cell responses in graft-versus-host disease^46^. The transcription factor RUNX1 is a member of the Runt-related transcription factor family of genes encoding the DNA-binding α-chain partners of the heterodimeric CBF complex^36,47^. It has been demonstrated that RUNX1 can mediate bone morphogenetic protein signaling in human DC maturation^48^ and is required for the development of Flt3^+^ DC progenitors and all mature DC lineages^37^. In androgen-dependent prostate cancer, the RUNX promoter is bound by EZH2 and negatively regulated by H3K27me3^38^. The interaction between EZH2 and RUNX1 was proven in our system. We observed that EZH2 deficiency enhanced the expression of RUNX1. EZH2-mediated regulation of immune responses induced by RUNX1 in DCs was demonstrated by inhibition of RUNX1 with its inhibitor Ro 5-3335 in the context of EZH2 deficiency both in vivo and in vitro. Inhibition of RUNX1 restored LPS-induced activation and DC-T cell responses in EZH2-deficient DCs, suggesting that EZH2 regulates the activation and immune functions dependent on RUNX1. However, according to the results, the effect of EZH2 deficiency in DCs was not linked to only RUNX1 expression. Other mechanisms, in addition to the mechanism involving RUNX1, may be involved. For example, the tumor necrosis factor (TNF) superfamily is a large family of molecules expressed by various immune cells and induced during the activation of the immune system. TNF superfamily members can influence DC biology, promoting homeostasis, maximizing T cell-DC encounters, and enhancing T-cell proliferation and cytokine secretion in the immune response. It was reported that the TNF superfamily ligands TNF^49^ and CD40 ligand^50^ play key roles in promoting the proliferation of bone marrow-derived progenitor cells and their differentiation into DCs. In previous research, it was shown that EZH2-catalyzed H3K27 trimethylation plays a key role in acute-on-chronic liver failure via a TNF-mediated pathway^29^. The results suggested that EZH2/H3K27me3 promoted the progression of liver failure and that TNF, along with downstream NF-κB and Akt signaling pathways, was manipulated by epigenetic modification with H3K27me3. Therefore, the effect of EZH2 deficiency on DCs may be correlated with the regulation of the TNF pathway in addition to the regulation of RUNX1 expression. It was also verified in vivo that Ro 5-3335 treatment promoted the progression of FHF in *Ezh2*^D−/−^ mice. However, whether the RUNX1 promoter is associated with EZH2, the regions occupied by EZH2 and the binding sites for EZH2 in the RUNX1 gene remain to be further confirmed by CHIP assays in the future.

Impressive studies have demonstrated the therapeutic potential of EZH2 inhibition, and numerous highly selective small-molecule inhibitors targeting EZH2 have been developed, some of which have entered clinical development^51,52^. With the aim of exploring the methyltransferase activity of EZH2 and histone modification, we selected GSK126 based on its high selectivity in inhibiting methyltransferase by competing with *S*-denosylmethionine without degrading EZH2^53^. GSK126 has been shown to ameliorate disease severity in acute-on-chronic liver failure by modulating TNF and overall inflammation^29^. The effect of GSK126 was also confirmed in the current study, suggesting its potential clinical application in FHF.

Overall, our study investigated the roles of EZH2 in the immune functions of DCs and the epigenetic mechanisms of EZH2 in FHF, suggesting that EZH2 promotes the development of liver failure in FHF, which was verified with *Ezh2*^D−/−^ mice and ameliorated using the inhibitor GSK126, via modulation of immune responses. Moreover, inhibition of EZH2 enhanced the expression of RUNX1, which functions as a downstream effector of EZH2 in DCs. This is the first study to provide direct experimental evidence proving the significant role of DC EZH2 in FHF, supporting the potential clinical applicability of pharmacological targeting of epigenetic modifications for the treatment of FHF.

## Supplementary information

Supplementary Table

Supplementary figure legends

Supplementary figure 1

Supplementary figure 2

Supplementary figure 3

Supplementary figure 4

Supplementary figure 5

Supplementary figure 6

Supplementary figure 7
