# Supplementary material for: Inhibition of EZH2 ameliorates bacteria-induced liver injury by repressing RUNX1 in dendritic cells
Source: Cell Death Dis. 2020 Dec 1;11(11):1024. doi: 10.1038/s41419-020-03219-w (PMC7708645; doi:10.1038/s41419-020-03219-w)
Supplement: Supplementary file 1 — Supplementary Table [file 41419_2020_3219_MOESM1_ESM.docx]

**Supplementary Table 1. Primer sequence**

| Name | Sequence |
| --- | --- |
| β-actin forward | 5’-TGTCCACCTTCCAGCAGATGT-3’ |
| β-actin reverse | 5’-AGCTCAGTAACAGTCCGCCTAGA-3’ |
| Ezh2 forward | 5’-CAACCCGAAAGGGCAACAAA-3’ |
| Ezh2 reverse | 5’-TCACCAGTCTGGATAGCCCT-3’ |
| TNF-α forward | 5’-GGTCTGGGCCATAGAACTGA-3’ |
| TNF-α reverse | 5’-CAGCCTCTTCTCATTCCTGC-3’ |
| IFN-γ forward | 5’-ATGAACGCTACACACTGCATC-3’ |
| IFN-γ reverse | 5’-CCATCCTTTTGCCAGTTCCTC-3’ |
| IL-6 forward | 5’-CACAGAGGATACCACTCCCAACA-3’ |
| IL-6 reverse | 5’-TCCACGATTTCCCAGAGAACA-3’ |
