# Supplementary material for: Inhibition of EZH2 ameliorates bacteria-induced liver injury by repressing RUNX1 in dendritic cells
Source: Cell Death Dis. 2020 Dec 1;11(11):1024. doi: 10.1038/s41419-020-03219-w (PMC7708645; doi:10.1038/s41419-020-03219-w)
Supplement: Supplementary file 2 — Supplementary figure legends [file 41419_2020_3219_MOESM2_ESM.docx]

**Supplementary Figure Legends**

**Supplementary Figure 1 Gating strategy used for DCs.** (A) MNCs isolated from livers were gated on the basis of size, granularity, singularity and expression of the leukocyte marker CD45, and CD11c^high^MHCII^high^ cells were identified.

**Supplementary Figure 2 Purity of sorted DCs.** (A) CD11c^+^ cells were positively sorted from liver MNCs using MACS CD11c microbeads and identified with flow cytometry. Gray shading indicates the isotype results.

**Supplementary Figure 3 Efficient deletion of the *Ezh2* gene in mice.**

(A) Genotyping PCR analysis of tail DNA from *Ezh2*^+/-^, *Ezh2*^+/+^, *Ezh2*^-/-^, and *CD11c*-cre mice.

**Supplementary Figure 4 Hepatic and splenic DC subsets in mice during FHF.** WT mice and *Ezh2*^D-/-^ mice were injected intravenously with *P. acnes* suspended in PBS. On day 7, LPS was injected to induce FHF. Spleens and livers were isolated 2 hours after LPS injection for analysis of the indicated cell populations (n=4 mice per group). MNCs were isolated from the liver and spleen. (A) The levels of CD103 and CD11b on CD45^+^ CD11c^+^ MHCII^+^ liver MNCs were analyzed by flow cytometry. (B) The levels of CD8 and CD11b on CD45^+^ CD11c^+^ MHCII^+^ spleen MNCs were analyzed by flow cytometry. Data are shown as the mean ± SEM of three independent experiments. ******P* < 0.05.

**Supplementary Figure 5 Changes in the weight of mice during FHF.** (A) The weights of *Ezh2*^D-/-^ mice were measured and recorded every day during the progression of FHF.

**Supplementary Figure 6 The absolute number of Tregs.** *Ezh2*^D-/-^ mice and WT mice were injected intravenously with *P. acnes* suspended in PBS. Livers and spleens were isolated from naïve, *Ezh2*^D-/-^ mice and WT mice on day 7. (A) MNCs isolated from the liver or spleen were stained for CD4 and Foxp3 and analyzed by flow cytometry. The number of CD4^+^ Foxp3^+^ Tregs was calculated (n=6 per group). Data are shown as the mean ± SEM of three independent experiments. ******P* < 0.05.

**Supplementary Figure 7 The effect of RUNX1 inhibition on T cell proliferation.** (A) Purified CD4^+^ T cells (3×10^5^ cells/well) from the spleen of naïve WT mice were cultured with 50 μM Ro 5-3335, and 72 hours later, T cell proliferation was assessed by analysis of CFSE dilution by flow cytometry.
