# Supplementary figures and images for: Inhibition of EZH2 ameliorates bacteria-induced liver injury by repressing RUNX1 in dendritic cells
Source: Cell Death Dis. 2020 Dec 1;11(11):1024. doi: 10.1038/s41419-020-03219-w (PMC7708645; doi:10.1038/s41419-020-03219-w)

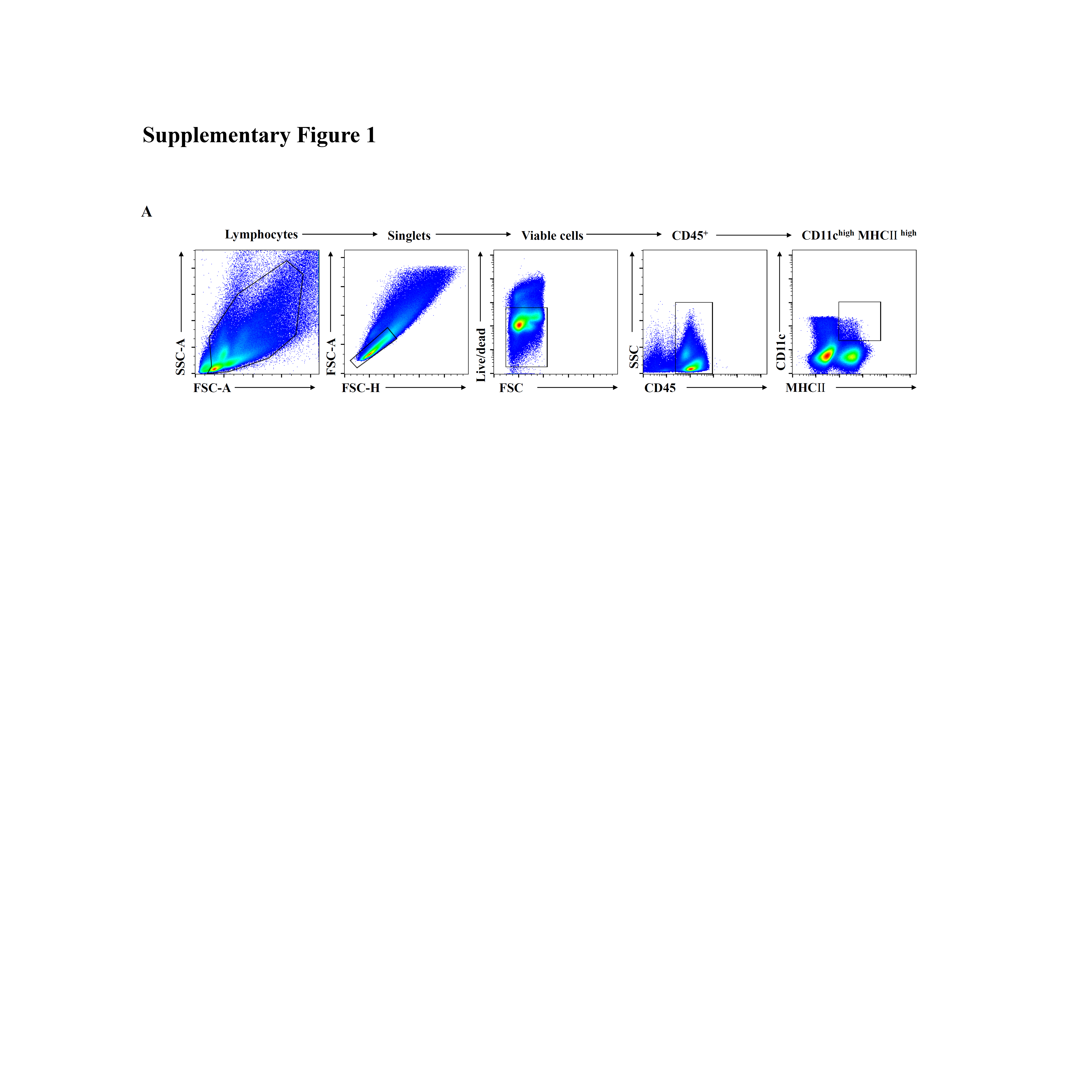

Supplement: Supplementary file 3 — Supplementary figure 1 [file 41419_2020_3219_MOESM3_ESM.tif]

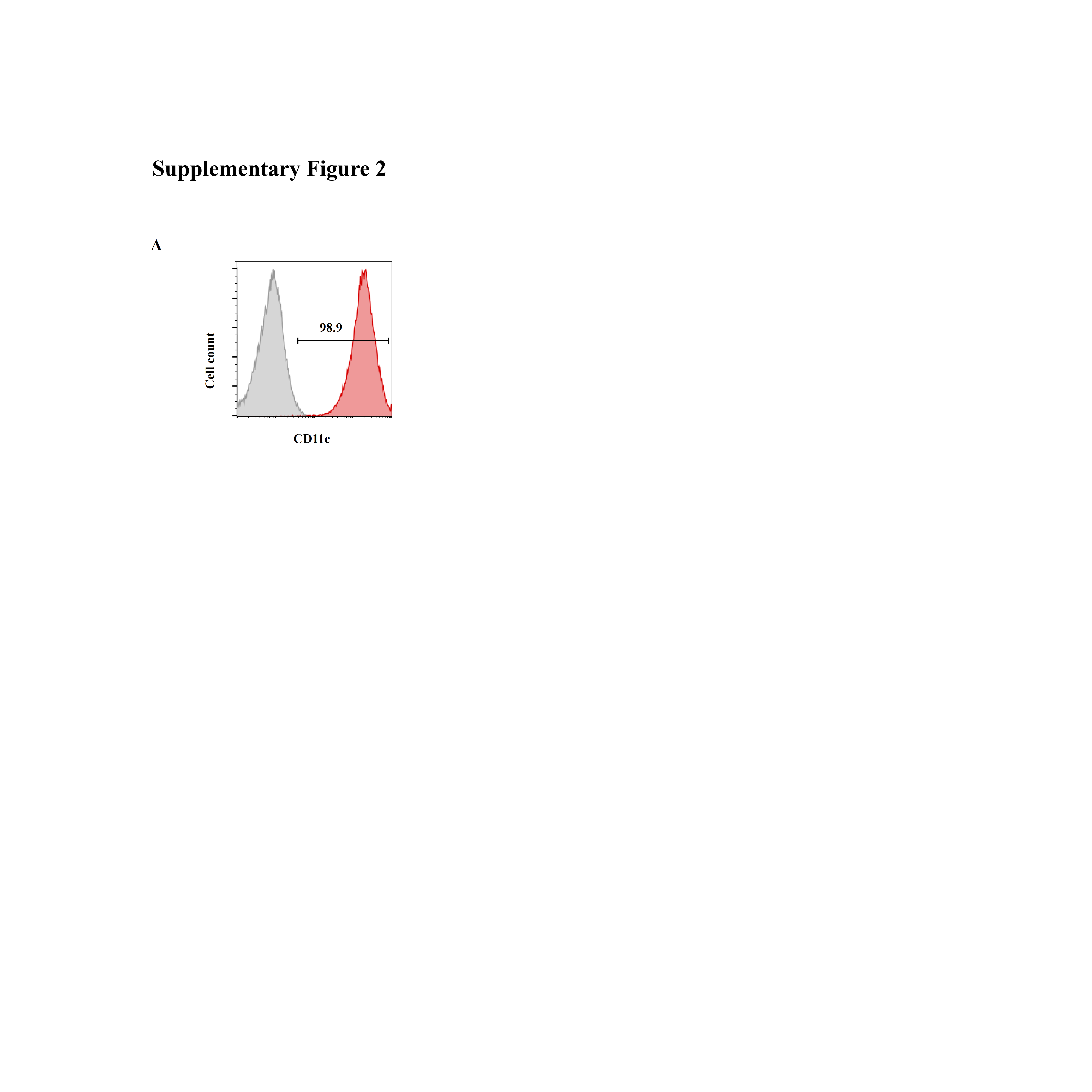

Supplement: Supplementary file 4 — Supplementary figure 2 [file 41419_2020_3219_MOESM4_ESM.tif]

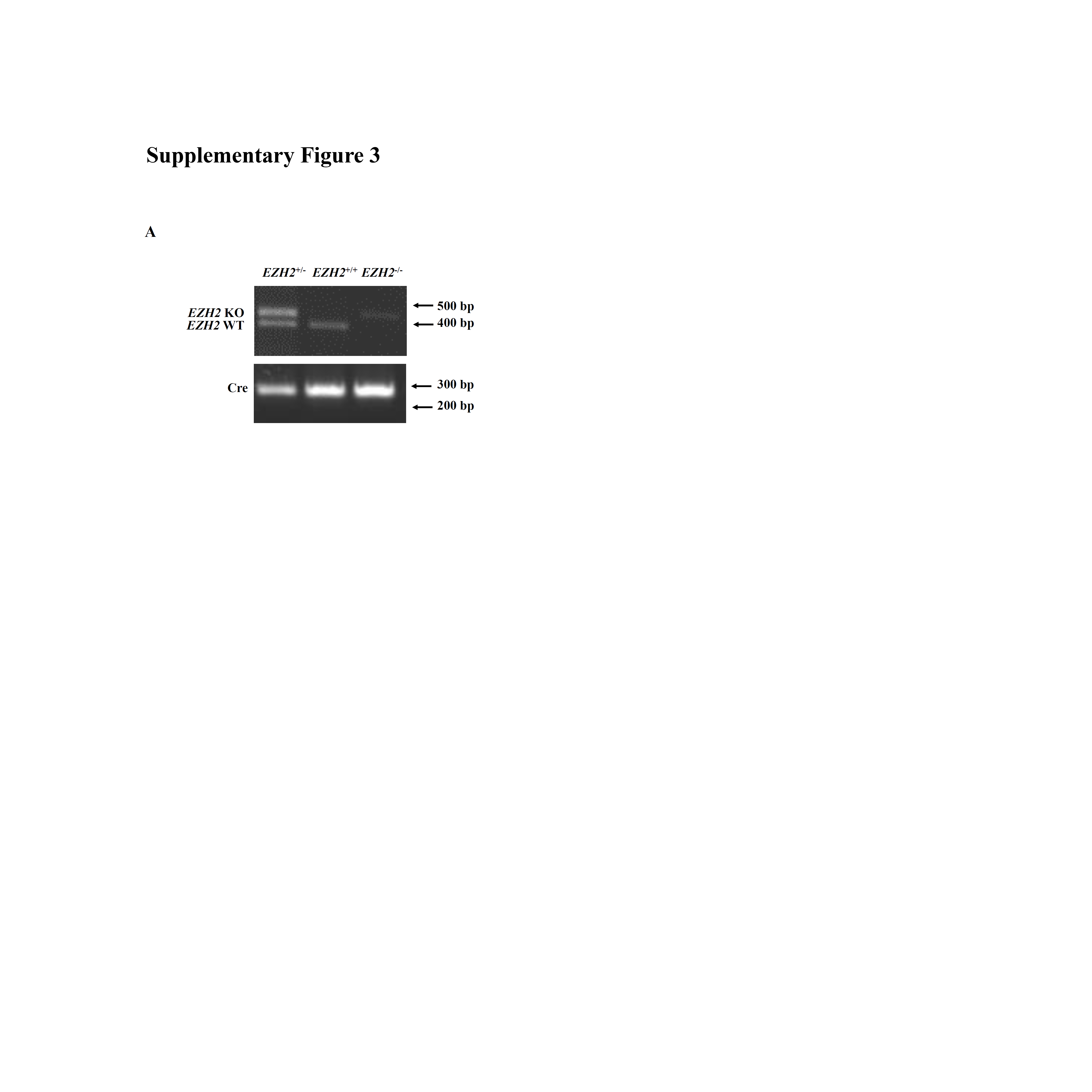

Supplement: Supplementary file 5 — Supplementary figure 3 [file 41419_2020_3219_MOESM5_ESM.tif]

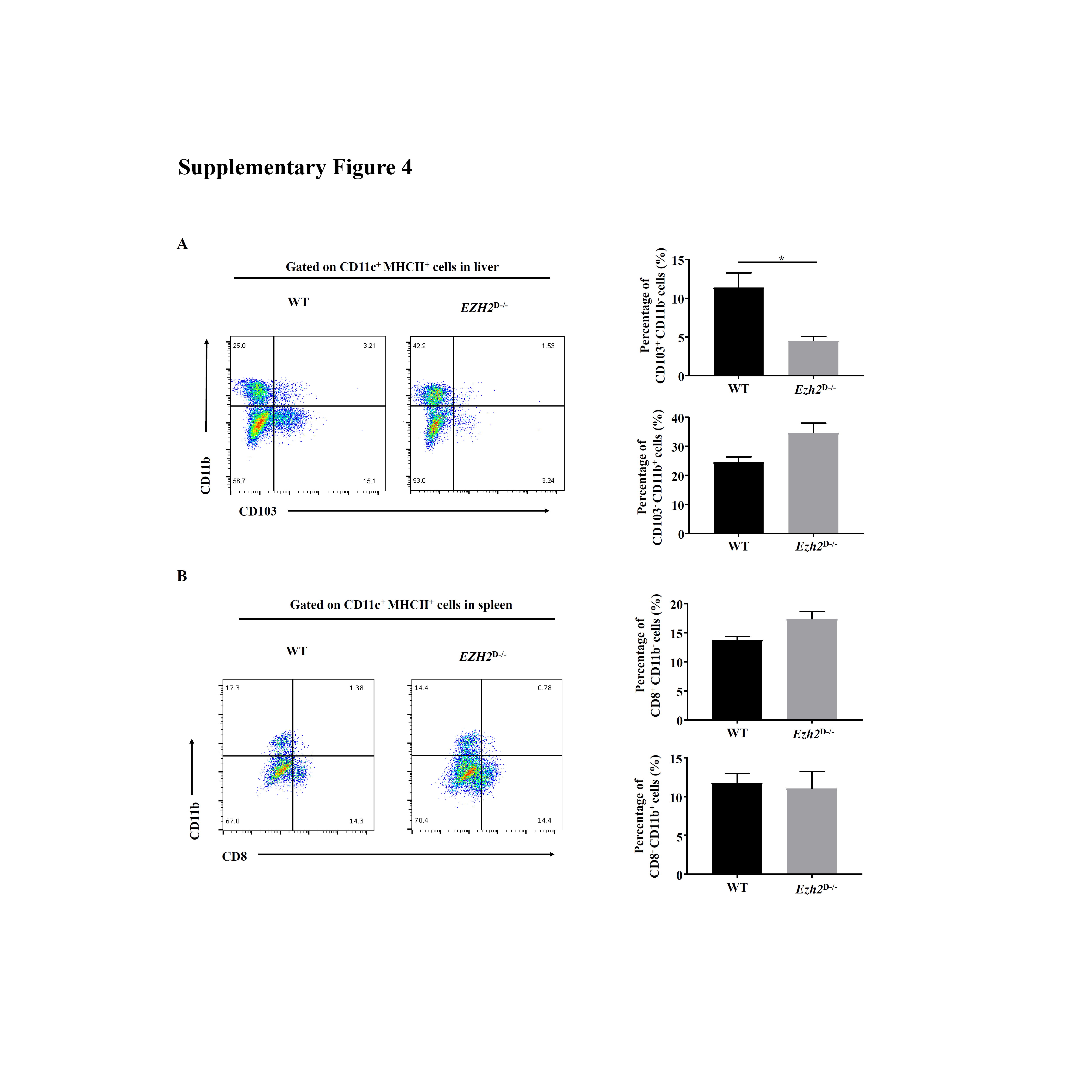

Supplement: Supplementary file 6 — Supplementary figure 4 [file 41419_2020_3219_MOESM6_ESM.tif]

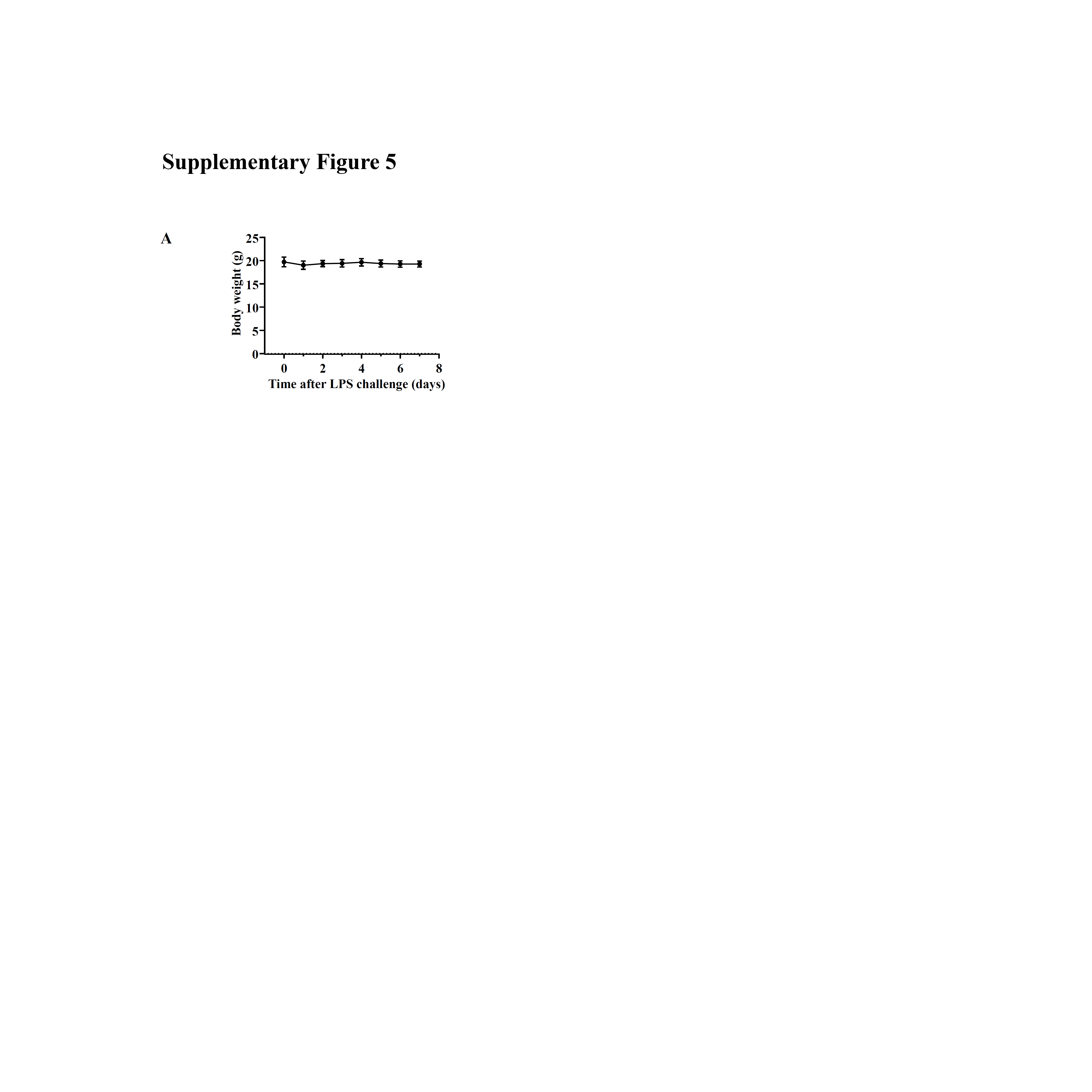

Supplement: Supplementary file 7 — Supplementary figure 5 [file 41419_2020_3219_MOESM7_ESM.tif]

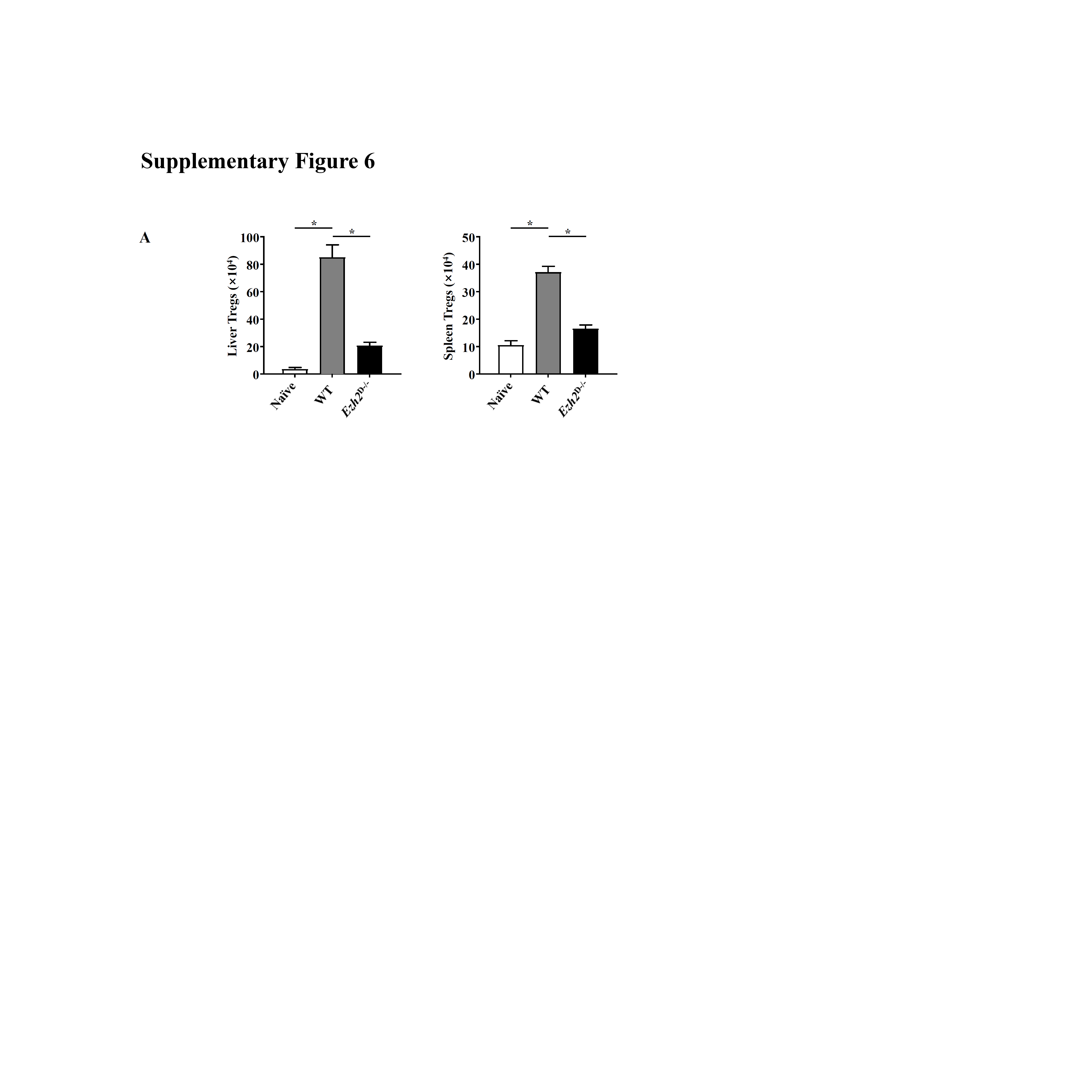

Supplement: Supplementary file 8 — Supplementary figure 6 [file 41419_2020_3219_MOESM8_ESM.tif]

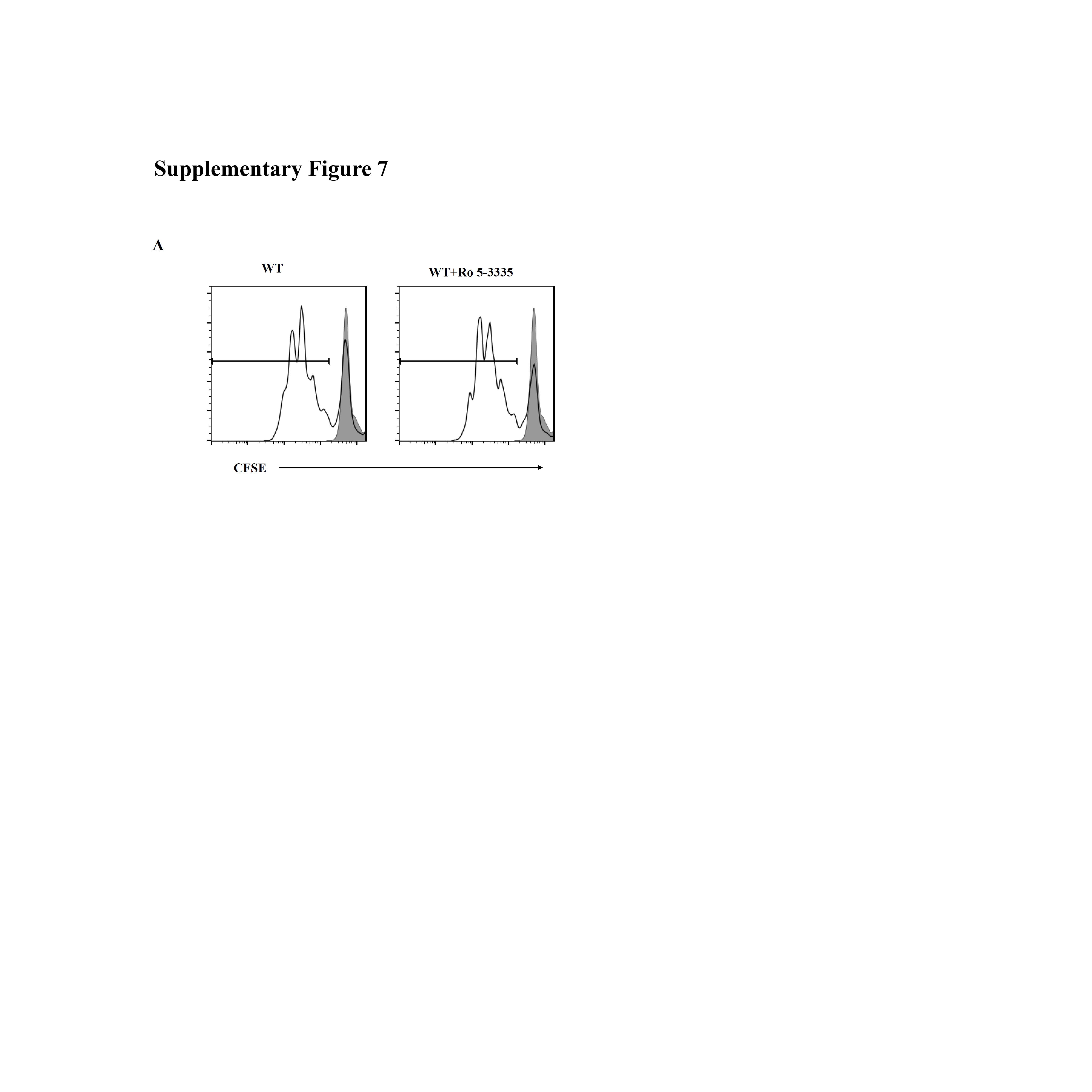

Supplement: Supplementary file 9 — Supplementary figure 7 [file 41419_2020_3219_MOESM9_ESM.tif]
